# Clinical and laboratory characteristics of penicilliosis marneffei among patients with and without HIV infection in Northern Thailand: a retrospective study

**DOI:** 10.1186/1471-2334-13-464

**Published:** 2013-10-05

**Authors:** Rathakarn Kawila, Romanee Chaiwarith, Khuanchai Supparatpinyo

**Affiliations:** 1 Department of Medicine, Faculty of Medicine, Chiang Mai University, Chiang Mai 50200, Thailand; 2 Research Institute for Health Sciences, Faculty of Medicine, Chiang Mai University, Chiang Mai, Thailand

**Keywords:** Penicilliosis marneffei, HIV-uninfected individuals, Epidemiology

## Abstract

**Background:**

Penicilliosis marneffei is increasingly observed in individuals without HIV infection. This study aimed to compare the clinical and laboratory features among HIV infected and uninfected individuals with penicilliosis marneffei.

**Methods:**

A retrospective cohort study was conducted between January 1, 2007 and December 31, 2011 at Chiang Mai University Hospital. We included individuals who were ≥15 years of age and presented with culture-proven *P. marneffei* infection.

**Results:**

116 HIV-infected and 34 HIV-uninfected patients were enrolled. Comparing to HIV-infected patients, HIV-uninfected patients were older; less likely to have fever, splenomegaly, and umbilicated skin lesions; more likely to have Sweet’s syndrome and bone and joint infections; had higher white blood cell count, platelet count, and CD4 cell count; had lower alanine transaminase (ALT); and less likely to have positive fungal blood cultures. The mortality rates were 20.7% and 29.4% among HIV infected and uninfected patients, respectively.

**Conclusions:**

Clinical manifestations of penicilliosis marneffei are different between patients with and without HIV infection. Physician’s awareness of this disease in HIV-uninfected patients may prompt the diagnosis and timely treatment, and can lead to a better outcome.

## Background

Systemic mycosis caused by *P. marneffei* is known to be endemic in South and Southeast Asia, and is a common human immunodeficiency virus (HIV)–associated opportunistic infection [1], ranking the third after tuberculosis and cryptococcal meningitis in northern Thailand [2]. HIV-infected individuals with CD4 cell count <100 cells/μL are at particular risk and make up the majority of infections in regions of endemicity [2,3]. It is also increasingly observed in immunocompromised individuals from non-endemic regions who have traveled to Southeast Asia [4] as well as those without HIV infection [5,6].

Previous small studies found that signs and symptoms of penicilliosis marneffei may be different in HIV-infected individuals compared to those without HIV infection [5,6]. These clinical characteristics, if existed, could lead to prompt diagnosis and timely treatment among HIV-uninfected individuals since disseminated penicilliosis marneffei, if left untreated, has a considerably high mortality [3]. This retrospective cohort study, with larger number of patients, aimed to compare the clinical and laboratory features among HIV infected and uninfected individuals with penicilliosis marneffei.

## Methods

### Study design and population

A retrospective cohort study was conducted between January 1, 2007 and December 31, 2011 at Chiang Mai University Hospital, a 1500-bed, tertiary-care hospital in Northern Thailand. Individuals included in this study were ≥15 years of age and had culture-proven *P. marneffei* infection. Data extracted from the medical records included demographic information (sex and age), clinical characteristics, laboratory findings, and clinical outcomes.

### Mycological methods

Blood samples were processed in the VersaTREK/REDOX (TREK diagnostic system, Cleveland, OH, USA) continuous monitoring blood culture system. Cultures of clinical specimens, including sputum, lymph node, tissue, and bone marrow were performed on Sabouraud’s dextrose agar at 25°C. Isolates visible within two to three days of incubation were subcultured on brain-heart infusion agar and incubated at 37°C [4,7].

Positive cultures for *P. marneffei* were characterized by dimorphic fungi that grew as a mold at 25°C and as yeast at 37°C. After 2 days, the mold colony was grayish white and downy or woolly. A unique characteristic of *P. marneffei* mold is the presence of a soluble red pigment that diffuses into the agar making the reverse side appears either pink or red [4,7].

This study was approved by the Faculty of Medicine, Chiang Mai University Ethical Committee.

### Statistical analysis

Clinical data were presented in numbers (%); mean and standard deviation (SD); and median and interquartile range (IQR) as appropriate. Comparison of demographic data and clinical characteristics between patients with and without HIV-infection was performed using Student’s t-test, Mann–Whitney U test, Chi-square test, or Fisher’s exact test as appropriate. Variables with a p-value <0.10 from the univariable analysis were then tested in multivariable models using forward stepwise procedures.

All statistical analyses were performed using Stata statistical software version 10.0 (Stata Statistical Software: Release 10.0, Stata Corporation, College Station, TX, 2007). A two-sided test at a p-value of <0.05 was used to indicate statistical significance.

## Results

### Demographic data and clinical characteristics

During the study period of 4 years, 153 patients presented with culture-proven penicilliosis marneffei; 116 were HIV-infected and 34 were HIV-uninfected. Three patients had unknown HIV status and were excluded from the analysis. Table 1 shows the comparison of demographic data and clinical characteristics between patients with and without HIV infection. Among 116 HIV-infected patients, the route of HIV transmission included heterosexual (105 patients, 90.5%), homosexual (nine patients, 7.8%), and intravenous drug users (two patient, 1.7%). Forty-two HIV-infected patients (36.2%) had previous history of opportunistic infections. The most common opportunistic infections were tuberculosis (23 patients, 54.8%) and *Pneumocystis jiroveci* pneumonia (12 patients, 28.6%).

**Table 1:** Clinical characteristics of penicilliosis marneffei among patients with and without HIV infection

**Variables**	**HIV-infected patients (N = 116)**	**HIV-uninfected patients (N = 34)**	**p-values**
Age (years)	39 (32, 48.5)	55 (50, 64)	<0.001
Female	37 (31.9%)	17 (50%)	0.068
Body weight (kilograms)	49 (42, 57)	48 (40, 52)	0.172
Underlying diseases*	116 (100%)	12 (35.3%)	<0.001
HIV	116 (100%)	0 (0)	
Diabetes	2 (1.7%)	3 (8.8%)	
Lymphoma	0	2 (5.9%)	
Colon cancer	0	1 (2.9%)	
Systemic lupus erythematosus	0	6 (17.7%)	
Mixed connective tissue disease	0	1 (2.9%)	
Myasthenia gravis	0	1 (2.9%)	
Receiving immunosuppressive drugs^†^	0	6 (17.7%)	<0.001
Previous opportunistic infections or co-infections	42 (36.2%)	31 (91.2%)	<0.001
*Cryptococcus neoformans*	3 (7.1%)	0	
*Mycobacterium tuberculosis*	23 (54.8%)	3 (9.7%)	
*Pneumocystis jiroveci*	12 (28.6%)	0	
Non tuberculous mycobacteria	2 (4.8%)	15 (48.4%)	
Cytomegalovirus	5 (11.9%)	0	
Herpes zoster virus	7 (16.7%)	0	
*Candida* spp.	2 (4.8%)	0	
*Isospora belli*	1 (2.4%)	0	
*Histoplasma capsulatum*	0	1 (3.2%)	
*Salmonella* spp.	0	8 (25.8%)	
*Rhodococcus equi*	0	1 (3.2%)	

Data are presented in number (%) or median (IQR).

*One patient may have ≥1 underlying diseases.

^†^See text.

Among 34 HIV-uninfected patients, 12 had underlying diseases, i.e., SLE receiving prednisolone and azathioprine (2); SLE and diabetes receiving prednisolone and mycophenolate (1); SLE receiving prednisolone (1); active SLE without immunosuppressive drugs (1); inactive SLE (1); mixed connective tissue disease receiving prednisolone (1); diabetes and myasthenia gravis receiving chemotherapy (1); lymphoma without chemotherapy (1); colon cancer without chemotherapy (1); and diabetes (2). Twenty-two patients (64.7%) had neither underlying diseases nor immunocompromised conditions. From March 2011 to March 2012, serum samples from 9 of these patients were tested and all were positive for autoantibody to interferon-ɤ using ELISA method. Thirty-one (91.2%) HIV-uninfected patients had prior history of or concomitant opportunistic infections; the most common was non-tuberculous mycobacteria (15 patients, 48.4%), followed by salmonellosis (eight patients, 25.8%).

The common clinical manifestations among all patients included fever (123 patients, 82%), cutaneous lesions (61, 40.7%), hepatomegaly (48 patients, 32%), and lymphadenopathy (50 patients, 33.3%). Patients infected with HIV were more likely to have fever and splenomegaly, whereas HIV-uninfected individuals were more likely to have bone and joint infections, i.e., arthritis, spondylodiskitis, and osteomyelitis. The median duration of symptoms before presenting to the hospital was one month and was not different between groups (Table 2).

**Table 2:** Clinical features of penicilliosis marneffei among patients with and without HIV infection

**Variables**	**HIV-infected patients (N = 116)**	**HIV-uninfected patients (N = 34)**	**p-values**
Fever	101 (87.1%)	22 (64.7%)	0.005
Lymphadenopathy	37 (31.9%)	13 (38.2%)	0.537
Hepatomegaly	42 (36.2%)	6 (17.6%)	0.059
Splenomegaly	25 (21.6%)	2 (5.9%)	0.042
Cutaneous lesions	47 (40.5%)	14 (41.2%)	1.000
Umbilicated lesions	45 (95.7%)	5 (35.7%)	
Sweet’s syndrome	0	5 (35.7%)	
Pustular psoriasis	1 (2.1%)	1 (7.1%)	
Chronic ulcer	1 (2.1)	0	
Subcutaneous nodule	0	1 (7.1%)	
Subcutaneous abscess	0	1 (7.1%)	
Panniculitis	0	1 (7.1%)	
Cough	32 (27.6%)	8 (23.5%)	0.826
Anemia	8 (6.9%)	0	0.199
Diarrhea	15 (12.9%)	2 (5.9%)	0.362
Arthritis/osteomyelitis	0	5 (14.7%)	<0.001
Duration of symptoms (days) (Median, IQR)	30 (14, 30)	30 (14, 60)	0.061

Laboratory findings differed between groups; HIV-infected individuals were more likely to have leukopenia, lower platelet count, transaminitis, and positive blood cultures compared with those without HIV infection. (Table 3) CD4 cell counts were available in 19 HIV-uninfected patients; the median CD4 cell count was 640 cells/mm^3^ (IQR 428, 833), which was significantly higher than that of HIV-infected patients.

**Table 3:** Laboratory findings of penicilliosis marneffei among patients with and without HIV infection

**Variables**	**HIV-infected patients (N = 116)**	**HIV-uninfected patients (N = 34)**	**p-values**
Hemoglobin (g/dL)	9.6 ± 2.1	9.7 ± 1.8	0.726
White blood cells (× 10^3^cells/ mm^3^)	4.1 (3.0, 5.9)	15.6 (10.1, 24.0)	<0.001
Platelet count (× 10^3^ cells/mm^3^)	119.5 (68.9, 218.0)	390.0 (176.0, 508.0)	<0.001
Creatinine (mg/dL)	0.9 (0.75, 1.2)	1.1 (0.8, 1.4)	0.168
CD4 cell count (cells/mm^3^)	14 (8, 40)	640 (428, 833)	<0.001
	(N = 105)	(N = 16)	
CD4%	3 (1, 6)	30 (28, 37)	<0.001
	(N = 105)	(N = 16)	
ALT (U/L)	43.5 (25.5, 69.5)	17.5 (14, 34)	<0.001
Albumin (g/dL)	2.5 (2.1, 3.2)	2.3 (1.9, 2.7)	0.164
Positive cultures			
Blood	89/116 (76.7)	16/34 (47.1)	0.001
Lymph node	21/31 (67.7)	7/13 (53.8)	0.496
BAL fluid	1/4 (25.0 )	4/10 (40.0)	1.000
Bone marrow	28/52 (53.8)	3/8 (37.5)	0.465
Skin	42/47 (89.4)	11/14 (78.6)	0.369
Sputum	3/32 (9.4)	1/8 (12.5)	1.000
Chest roentgraphy	N = 33	N = 16	
Normal	9 (27.3%)	1 (6.3%)	0.135
Abnormal			
Interstitial infiltration	10 (30.3%)	0	
Alveolar infiltration	7 (21.2%)	7 (43.8%)	
Pleural effusion	4 (12.1%)	7 (43.8%)	
Interstitial and alveolar	0	1 (6.3%)	
Cavitary lesion	3 (9.1%)	0	

Data are presented in number (%), median (IQR), or mean ± standard deviation.

*Abbreviations*: *ALT* Alanine aminotransferase, *BAL* Bronchoalveolar lavage.

Multivariate analysis showed that factors associated with penicilliosis marneffei in patients without HIV infection included age ≥40 years (OR 7.63, 95% CI 2.05, 28.43), WBC ≥5000 cells/mm^3^ (OR 9.74, 95% CI 3.03, 31.27), and alanine transaminase <40 U/L (OR 3.68, 95% CI 1.32, 10.31).

### Treatment and outcome

Fourteen HIV-infected and 4 HIV-uninfected patients did not receive antifungal treatment since the diagnosis of penicilliosis marneffei could not be made during hospitalization. The majority of patients in this study were treated with intravenous amphotericin B deoxycholate followed by itraconazole (Table 4). The dosage and duration of treatment among HIV-infected patients were intravenous amphotericin B deoxycholate 0.6-1.0 mg/kg/day for 2 weeks, followed by oral itraconazole 400 mg/day for 8 to 10 weeks. After completion of treatment, all HIV-infected patients received oral itraconazole 200 mg/day for secondary prophylaxis. For HIV-uninfected patients, the duration of treatment varied widely with the median of 180 days (IQR 84, 180). HIV-infected individuals were more likely to receive a shorter course of treatment than those without HIV infection.

**Table 4:** Treatment outcome of penicilliosis marneffei patients with and without HIV infection

**Variables**	**Number of patients (%)**		**p-values**
	**HIV-infected patients (N = 116)**	**HIV-uninfected patients (N = 34)**	
Treatment received	102 (87.9)	30 (88.2)	1.000
Medication			0.823
-Amphotericin B then itraconazole	71 (69.6)	20 (66.7)	
-Itraconazole	31 (30.4)	10 (33.3)	
Outcome			0.351
-Recovery	92 (79.3)	24 (70.6)	
-Dead	24 (20.7)	10 (29.4)	
Treatment duration (days) (median, IQR)	84 (84, 90)	180 (84, 180)	<0.001

The mortality rate was 20.7% (24 patients) and 29.4% (ten patients) among HIV-infected and uninfected patients, respectively (p-value = 0.285). Causes of death in 10 HIV-uninfected patients included penicilliosis marneffei (1), mixed infection with *Salmonella enteritidis* (1), and hospital-acquired infections (8). Among the 24 survivors, five patients had non-tuberculous mycobacterial infection at a median duration of 4 months after diagnosis of penicilliosis marneffei. Nineteen patients had no other opportunistic infections after completion of treatment for penicilliosis marneffei.

## Discussion

Penicilliosis is a common but serious opportunistic fungal infection in Southeast Asian AIDS patients, although it is now increasingly observed in individuals who are not infected with HIV [5,6]. Since penicilliosis marneffei has been considered a rare entity in non-HIV individuals, making diagnosis in these patients is usually difficult and delayed [6]. The two previous studies from Hong Kong [5] and China [6] had tried to distinguish the clinical presentations between patients with and without HIV infection; however, many distinct clinical characteristics, if existed, might be missed due to the small sample size.

In this study, clinical manifestations among HIV infected patients were similar to those in other previous reports [1,4,5]. The majority of HIV-infected patients with penicilliosis marneffei presented with fever. However, anemia, hepatomegaly, lymphadenopathy, and skin lesions were less commonly observed in our study than in other reports [1,4,7,8]. In addition, comparing to the report from our institution in 1990s, we found that less patients presented with skin lesions (85.1% in 1990s vs. 40.5% in this study) and lymphadenopathy (83.8% in 1990s vs. 31.9% in this study) [7], which could be explained by the fact that, with these clinical characteristics, diagnosis could be easily made by physicians in the community hospitals and patient referral to the tertiary care hospital was unnecessary. Therefore, those presented or referred to our hospital recently may not have some characteristic clinical features commonly observed in the past.

In our study, 61.8% of patients without HIV infection who presented with penicilliosis marneffei had no recognized underlying diseases, comparing to 54.4% and 14.3% in the reports from China and Hong Kong, respectively [5,6]. Although we did not performed a complete immunological study to identify all possible immunological abnormalities in these patients, the CD4 cell counts that were available in 19 patients were apparently higher than those in HIV-infected individuals. This could be assumed that cell mediated immune deficiency in these patients was not from CD4 lymphocytopenia like in HIV infected patients. After the recent report by Browne et al. that autoantibody to interferon-ɤ is associated with the new clinical syndrome of adult-onset immunodeficiency in East Asian population [9], serum samples from 9 patients were tested and found positive for autoantibody to interferon-ɤ. This may be the cause of cell-mediated immunity defect in our patients without HIV infection.

Previous studies from China showed that HIV-uninfected patients (N = 11) had longer duration of illness before the diagnosis was made than that in HIV-infected patients (N = 22) (180 days vs. 45 days). These patients were also more likely to have intermittent fever, subcutaneous nodules and abscesses, generalized lymphadenopathy, bone ache, leukocytosis, and normal CD4/CD8 ratio (>0.5) [6]. In contrast, HIV-infected patients were more likely to have persistent fever, molluscum-like skin lesions, and positive blood cultures. The other study from Hong Kong comparing patients with (N = 8) and without (N = 7) HIV infection did not show any statistical difference due to small number of patients [5].

To our knowledge, this is the largest series from a single hospital to compare clinical manifestations of penicilliosis marneffei among HIV infected and uninfected patients. We could demonstrate that HIV-uninfected patients were older, more likely to have bone and joint infections, and had higher CD4 cell count, whereas HIV-infected patients were more likely to have fever, splenomegaly, leukopenia, low platelet count, elevation of alanine transaminase, and positive blood cultures. HIV-infected patients were also more likely to have umbilicated skin lesions, whereas HIV-uninfected patients were more likely to have reactive skin diseases such as Sweet’s syndrome. In contrary to the previous study from China [6], the median duration of illness in this study was not different between the two groups.

Many patients in both groups had previous history of or concomitant opportunistic infections that are usually the indicators of defect in cell-mediated immunity. However, penicilliosis marneffei was the first presenting opportunistic infection in 63.8% of HIV-infected patients, but in only 8.8% of HIV-uninfected individuals. Opportunistic infections in these 2 groups were also different; tuberculosis (54.8%) and *Pneumocystis jiroveci* pneumonia (28.6%) were more common in HIV-infected patients, whereas non-tuberculous mycobacterial infection (48.4%) and salmonellosis (25.8%) were more common in HIV-uninfected patients.

The majority of patients in this study received treatment for *P. marneffei* infection although the duration of treatment was significantly longer in HIV-uninfected patients. While guidelines for treatment of penicilliosis marneffei in HIV-infected patients were established [10], there are no standard recommendations regarding the appropriate duration of treatment and prophylaxis of penicilliosis marneffei among HIV-uninfected patients. Our anecdotal experience revealed that relapse was more common in patients who received shorter the duration of treatment.

The overall mortality rate of patients with penicilliosis marneffei was 22.7%. The mortality rate identified in this study was lower than other reports of both HIV-infected and uninfected patients [1,5,6]. We could not demonstrate a significant difference of mortality rate between patients with or without HIV infection (20.7% and 29.4%, p = 0.285).

This study has several limitations. First, some data were missing due to a nature of retrospective study. Second, due to a small number of HIV-uninfected patients, some different clinical characteristics between the 2 groups may not be captured.

## Conclusions

Penicilliosis marneffei is endemic in Southeast Asia. The incidence of this disease in individuals without HIV infection is recently increasing. Some clinical features in patients with and without HIV infection are different. Physician’s awareness of this disease in HIV-uninfected patients may prompt the diagnosis and timely treatment, and can lead to a better outcome.

## Competing interest

The authors declare that they have no competing interest.

## Authors’ contributions

RK participated in data collection, performed the statistical analysis, and draft the manuscript. RC participated in the design of the study, performed the statistical analysis, and drafted the manuscript. KS revised manuscript critically for important intellectual content. All authors read and approved the final manuscript.

## Pre-publication history

The pre-publication history for this paper can be accessed here:

http://www.biomedcentral.com/1471-2334/13/464/prepub
